# Prevalence of Malnutrition and Contributing Factors in Children With Cerebral Palsy in Malawi: A Case‐Control Study

**DOI:** 10.1111/mcn.70260

**Published:** 2026-10-29

**Authors:** Bente van der Meijden, Natasha Lelijveld, Alinafe Chisulanda Kusate, Lara Chikuse, Molly Margot Mascini, Wieger Voskuijl

**Affiliations:** ^1^ Amsterdam UMC, location University of Amsterdam, Amsterdam Centre for Global Child Health Emma Children's hospital Amsterdam the Netherlands; ^2^ Department of Family Medicine Kamuzu University of Health Sciences Blantyre Malawi; ^3^ Emergency Nutrition Network (ENN) Kidlington UK; ^4^ Department of Physiotherapy Mangochi District Hospital Mangochi Malawi; ^5^ Department of nutrition Mangochi District Hospital Mangochi Malawi; ^6^ Department of Global Health, Amsterdam Institute for Global Health and Development, Amsterdam UMC Location University of Amsterdam Amsterdam the Netherlands; ^7^ Department of Paediatrics and Child Health Kamuzu University of Health Sciences Blantyre Malawi

**Keywords:** children with disabilities, CP (Cerebral Palsy), growth disorders, LMIC, Malawi, malnutrition, undernutrition

## Abstract

Children with cerebral palsy (CP) are at increased risk of malnutrition, yet no population‐level data exist for this group in Malawi. This study aimed to estimate the prevalence of undernutrition among children with CP in Malawi compared to peers without CP, and to explore contributing factors. This cross‐sectional case‐control study was conducted in Mangochi District, Malawi. A total of 260 children aged 2–18 years with clinician‐confirmed CP were enroled alongside 259 age‐ and sex‐matched controls. Nutritional status was assessed using standard anthropometric measures. Feeding difficulties and household food security were captured using existing tools. Linear regression examined factors associated with weight‐for‐age (WAZ) and BMI‐for‐age z‐scores (BMIZ). Children with CP had significantly worse nutritional status across all anthropometric indicators compared to controls. Stunting (90.9% vs. 45.6%), underweight (81.5% vs. 33.2%), and wasting (42.2% vs. 12.3%) were substantially higher in the CP group (all *p* < 0.05). Feeding dependency was prevalent in 64.7% of children with CP versus 14.5% of controls. Severe motor impairment (GMFCS IV–V), spastic CP subtype, and greater feeding difficulties, were independently associated with lower WAZ and BMIZ scores. In conclusion, malnutrition is highly prevalent among children with CP in rural Malawi, far exceeding national and regional estimates. Disability‐inclusive nutrition guidelines, caregiver feeding support, and integration of CP‐specific care into existing nutrition programmes are urgently needed.

## Key messages

- Children with cerebral palsy in rural Malawi face an exceptionally high burden of malnutrition, with stunting (90.9%), underweight (81.5%) and wasting (42.2%) rates far exceeding national averages and those of matched peers without CP.
- Feeding difficulties, severe motor impairment, and household food insecurity are key contributors to poor nutritional outcomes in this population.
- Despite high rates of prior enrolment in outpatient therapeutic programmes, substantial undernutrition persists, highlighting the inadequacy of standard nutrition protocols for children with CP.
- Disability‐specific nutrition guidelines, caregiver feeding support, and integration of CP‐sensitive care into existing nutrition and rehabilitation programmes are urgently needed in Malawi and similar low‐resource settings.

## 1 Introduction

Cerebral palsy (CP) is the most common physical disability in childhood, characterised by permanent disturbances of movement and posture arising from a non‐progressive brain injury occurring in early life(Donald et al. 2015). According to a recent scoping review of African paediatric populations, prevalence estimates of CP in the region vary considerably, ranging from 0.8 to 10 per 1000 children, depending on study setting, methodology, and population sampled (Murugasen et al. 2024). This is substantially higher and more variable than the 1.6–2.9 per 1000 typically reported in high‐income countries (HIC)(Khandakar et al. 2015). Children with CP are particularly vulnerable to malnutrition(Fogarasi et al. 2022; Srishti and Renuka 2015), even when compared to other disabilities(Makhoul et al. 2025). This is driven by feeding and swallowing difficulties (dysphagia), reduced dietary intake, recurrent infections, and inadequate access to specialised care(Groce et al. 2014; Hume‐Nixon and Kuper 2018; L. Jahan et al. 2023). A recent systematic review and meta‐analysis estimated a pooled malnutrition prevalence of 59.7% among children with CP across sub‐Saharan Africa (SSA), with individual studies ranging from 37% to 90% (Chanie et al. 2024). Undernutrition contributes substantially to morbidity and mortality, particularly in low‐ and middle‐income countries (LMIC), where the combination of severe motor impairment and undernutrition has been associated with a significant increase in mortality risk compared with children without CP (Kerac et al. 2014; Kuper and Heydt 2019; Lelijveld et al. 2020; Namaganda et al. 2020). This is in stark contrast with the life expectancy of individuals with CP living in HIC, where most individuals live far into adulthood(Haak et al. n.d.).

The relationship between disability and nutrition is complex and bidirectional: malnutrition can exacerbate underlying neurological impairment, hinder motor and cognitive development, and increase susceptibility to infection, while disability itself can heighten nutritional vulnerability through multiple biological, functional, and social pathways(Dan 2016; Groce et al. 2014; Kerac et al. 2014; Novak et al. 2012). Undernutrition frequently results from oropharyngeal dysfunction affecting chewing and swallowing and prolonged mealtimes reflecting poor oral–motor control (Groce et al. 2014; Kerac et al. 2014). Feeding dependency is common, with approximately 22%–27% of children requiring full assistance during meals, and more in the moderate/severe CP group(Nyman et al. 2025; Schepers et al. 2022), placing considerable physical and emotional strain on caregivers. This burden, combined with fear of choking, limited time, uncertainty about appropriate feeding strategies, and subsequent mealtime fatigue, can lead to compromised feeding practices such as reduced feeding frequency. Additionally, this can lead to smaller or less nutritious meals, or force‐feeding, and contribute to the risk of undernutrition (Arvedson 2013; Polack et al. 2018; Yousafzai et al. 2003). These challenges further interact with broader social determinants of health—including poverty, stigma, and limited access to rehabilitation or inclusive health services—that compound the risk of undernutrition in this population (Groce et al. 2013, 2014; Marchand et al. 2006; Unicef 2022).

In an LMIC like Malawi, children with disabilities face substantial structural and socio‐economic challenges that heighten their vulnerability to poor health and nutritional outcomes. Community‐based (nutritional) rehabilitation and social support services remain scarce, and families frequently experience significant financial strain, limited access to healthcare, and high caregiving demands(Barlindhaug et al. 2016; Harrison et al. 2020). Recent data from our group from Malawi indicate markedly reduced quality of life among children with CP and their caregivers, with particularly low scores in domains related to pain, disability impact, and family health—reflecting the cumulative burden of caregiving in a context of widespread unemployment and household food insecurity (van der Meijden et al. 2026; van der Meijden, Wedda, et al. 2026). Although population‐level data on malnutrition among children with disabilities are lacking, the nutritional status of the general population in Malawi underscores the broader context of risk. 37.6% of children under five are stunted nationally(National Statistical Office 2025). This has prompted a formal national commitment by the Ministry of Health to reduce stunting prevalence to 33% by 2030, with special emphasis on inclusive and gender sensitive programmes, reflecting growing recognition of the scale of the problem(Global Nutrition report 2025).

Despite CP being recognised as the leading cause of childhood physical disability in the country (Tataryn et al. 2017), no national data exist on the nutritional status of this population, and the country lacks disability‐specific nutrition guidelines or structured feeding support services(Engl et al. 2022). The regional evidence base is itself limited: a recent review identified only 16 studies across nine SSA countries, none of them Malawi, and all were cross‐sectional designs. The authors were unable to assess the causes of malnutrition in this population and identified this as a priority for further research(Chanie et al. 2024). Previous research from Malawi on post‐discharge outcomes has documented persistent growth faltering and elevated mortality among children with disabilities, emphasising the need for context‐specific evidence to guide clinical and public health interventions(Kerac et al. 2014; Lelijveld et al. 2020).

To address this knowledge gap, the present study aims to: (1) estimate the prevalence of undernutrition among children and adolescents with CP in the Malawian setting, compared to an age‐ and sex‐matched cohort without CP; and (2) identify the child‐, caregiver‐ and household‐level factors associated with poorer nutritional status within the CP group, including motor severity, feeding difficulties, feeding dependency and household food insecurity.

## 2 Methods

### 2.1 Study Design

This was a cross‐sectional study comparing quantitative data between children with cerebral palsy (CP) and an age‐ and sex‐matched control group without CP.

### 2.2 Study Setting and Participants

The study was conducted in Mangochi District, a lakeshore region in southern Malawi, with 1.3 million residents in 2023. The district experiences severe poverty, with 64% of residents living below the national poverty line compared to 51% nationally(National Statistical Office 2023). While earlier national estimates reporting a stunting prevalence of 35,5% (2016)(Globalnutritionreport n.d.), more recent data from 2024 Malawi Demographic and Health Survey (DHS), confirms that undernutrition even more prevalent. Stunting affects 40,6% in Mangochi district and wasting 1.2%, compared with 37.6% and 2.0% nationally (National Statistical Office 2025). This reflects a context of widespread nutritional vulnerability in which children with disabilities may face additional risks.

Two trained research assistants enroled participants between February 2023 and September 2024. Inclusion criteria comprised children aged 2–18 years with clinician‐confirmed CP diagnoses whose primary caregivers demonstrated fluent Chichewa comprehension. Exclusion criteria included unclear diagnoses or unavailable primary caregivers. Children with CP were identified and enroled from three settings: the outpatient multidisciplinary CP clinic, home visits, and the paediatric inpatient ward. The multidisciplinary CP clinic at Mangochi District Hospital (MDH) is a weekly outpatient clinic with three associated outreach clinics, offering routine care through rehabilitation, nutritional care, medical care and caregiver health education. Children recruited in this setting were therefore attending scheduled follow‐up rather than presenting with acute illness, and were in generally stable health at the time of assessment. Only children recruited from the inpatient ward were acutely unwell. Controls were recruited exclusively through door‐to‐door enquiries in the same geographic communities, selecting the first eligible child encountered. Each control was matched to a child with CP on age (± 2 months) and sex. Children who were hospitalised at the time of recruitment were not eligible as controls. If eligible and they agreed to participate, primary caregivers provided written informed consent or, if unable to write, gave thumbprints with witness verification (Figure 1).

**Figure 1:**
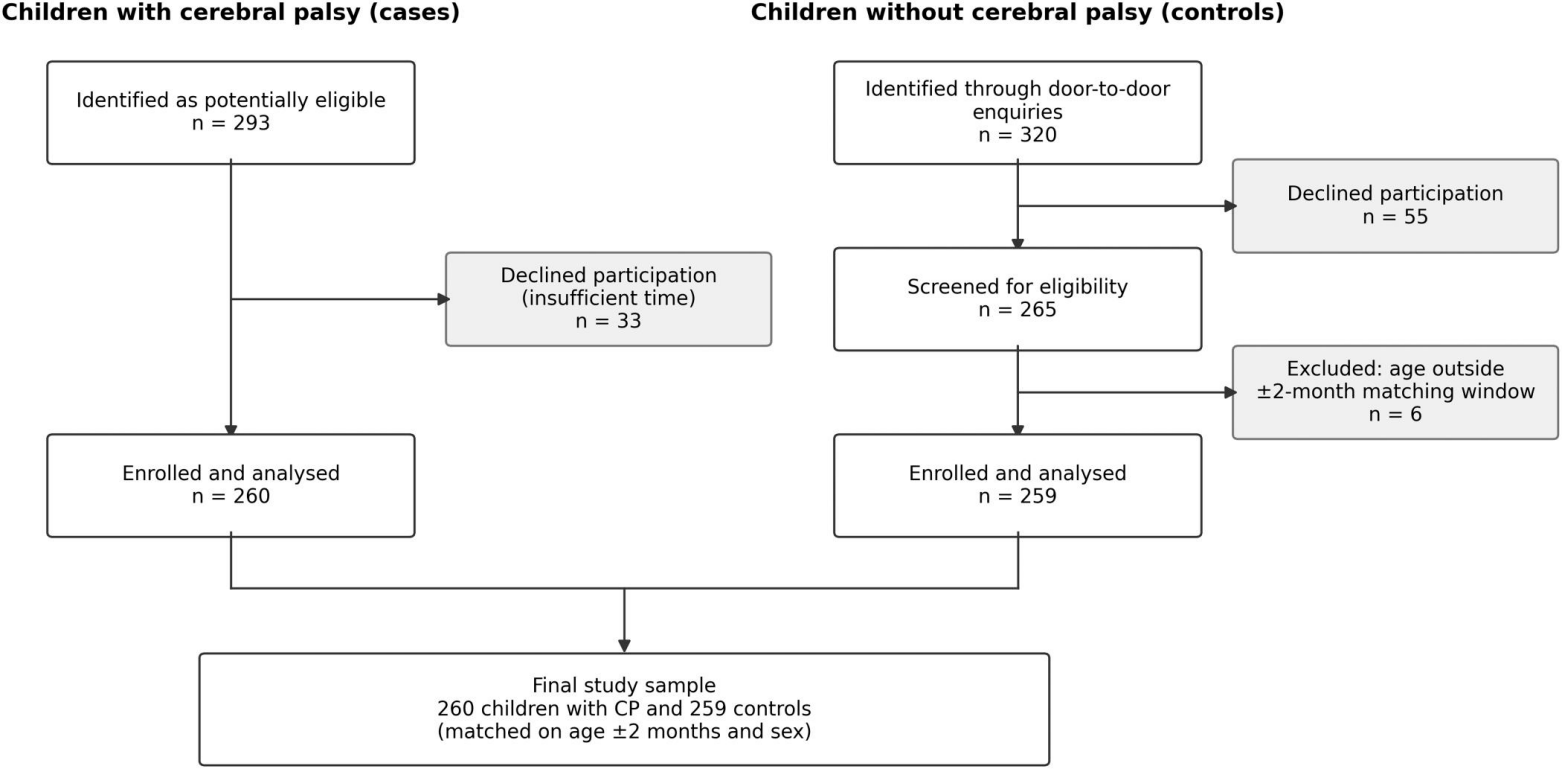
Flow diagram of participant recruitment and enrolment of children with cerebral palsy and age‐ and sex‐matched controls.

### 2.3 Data Collection

A structured questionnaire was used to collect data from both the child with CP (e.g. socio‐demographics, severity of CP, feeding difficulties, anthropometry) and the caregiver (socio‐demographics, economic status). Severity of CP was assessed using the Gross Motor Function Classification System (GMFCS), a standardised tool used to classify the severity of motor function limitations (Wood and Rosenbaum 2000). Feeding difficulties were assessed using a translated version of a 5‐point Likert scale questionnaire previously used and described by Polack et al.(Polack et al. 2018). Psychometric properties of this questionnaire were Cronbach's alpha of 0.8 and item‐total correlations above 0.7 (Polack et al. 2018). We added questions on medical history related to undernutrition and an extra question on 24 h food intake, coded against 13 food groups. This questionnaire can be found in Appendix 1.

To collect data about feeding practices, the household food security (HFS) questionnaire was used. The HFS section was adapted from the Food Insecurity Experience Scale (FIES), a validated 8‐item tool assessing food insecurity across diverse contexts and LMIC settings(Ballard et al. 2013, 2014; Cafiero et al. 2017). A total score for each participant was derived by adding the responses to a maximum of 8. A categorical variable defining food insecurity was created with a score of 0–3, 4–6 and 7–8 defined as low, moderate and severe food insecurity, respectively. All questionnaires were pre‐tested for clarity and cultural relevance.

### 2.4 Anthropometric Measurements

Nutritional status was assessed through standard anthropometric procedures:

1. Weight
  Measured using a SECA 310 hanging scale (max. 25 kg) (Seca Vogel & Halke GmbH & Co., Hamburg, Germany), or SECA 761 standing scale (Seca Vogel & Halke GmbH & Co., Hamburg, Germany). For children over 25 kg unable to stand, weight was estimated by subtracting caregiver's weight from the combined weight of caregiver and child(Malawi Ministry of Health n.d.). Scales were regularly calibrated; weights were recorded to the nearest 0.1 kg.
2. Height
  Height was measured using UNICEF height boards. For children unable to stand, recumbent length was measured. In case of significant musculoskeletal deformities (such as kyphosis, scoliosis or notable deformities in lower‐limb flexion) or severe spasticity, height estimates were predicted from the knee height. Knee height was measured twice, in seated position recorded to the nearest 0.1 cm, and converted to total height using the Stevenson equation, following the recommendations of the European Society for Paediatric Gastroenterology Hepatology and Nutrition (ESPGHAN)(European Society of Paediatric Gastroenterology, Hepatology, and Nutrition, ESPGHAN 2019). This applied to 11 children (4.3%). All height measurements were taken twice and recorded to the nearest 0.1 cm.
3. Mid‐Upper Arm Circumference (MUAC)
  MUAC was measured using UNICEF tapes on the left arm, recorded to the nearest 0.1 cm, and repeated for reliability, according to international standards(World Health Organisation 2013).

### 2.5 Data Management and Statistical Analysis

Data were entered into Microsoft Excel and analysed using IBM SPSS Statistics version 27. Data files were encrypted and password‐protected, with access restricted to study personnel only.

Anthropometric z‐scores were generated using World Health Organisation (WHO) Anthro (for children < 5 years) and AnthroPlus (for children ≥ 5 years) (WHO n.d.), based on the WHO growth standards(de Onis et al. 2007; WHO Multicentre Growth Reference Study Group 2006). Biologically implausible value flags were not applied. Extreme z‐scores are expected in children with cerebral palsy, particularly for height‐for‐age, where severe stunting produces values beyond conventional flagging limits; excluding these would have systematically removed the most severely affected children and biased estimates towards the null. All values were therefore retained, after review against source records to exclude data‐entry error.

Undernutrition indicators—stunting, wasting, underweight and thinness—were used to classify nutritional status. Stunting was defined as height‐for‐age z‐score (HAZ) < −2 (2–18 years); wasting (for < 5 years only) was defined as weight‐for‐height z‐score (WHZ) < −2 or MUAC‐for‐age z‐score (MUACZ) <−2. Thinness was defined as body mass index‐for‐age z‐score (BMIZ) < − 2 for children aged 2–18 years, and underweight was defined as weight‐for‐age z‐score (WAZ) <−2 (children < 10 years only). These age ranges reflect the ages over which each index is applicable rather than the age range of the sample, which spanned 24–215 months. The severity of undernutrition was classified based on z‐score thresholds as normal (≥−2), moderate (<−2 and ≥−3), and severe (<−3). In addition, age‐adjusted MUAC cut‐offs were used to report wasting levels as follows: for ages 6‐59 months: normal/no malnutrition (≥ 12.5 cm), moderate malnutrition (< 12.5 cm and ≥ 11.5 cm), and severe malnutrition (< 11.5 cm); and for ages 60‐–120 months: normal/no malnutrition (≥ 14.5 cm), moderate malnutrition (< 14.5 and ≥ 13.5 cm), and severe malnutrition (< 13.5 cm) (de Onis et al. 2007; WHO Multicentre Growth Reference Study Group 2006). Differences in wasting prevalence between age groups were assessed using Fisher's exact test.

Descriptive statistics were computed for all variables. GMFCS levels were regrouped for analysis as follows: mild CP (GMFCS levels I and II), moderate CP (GMFCS level III) and severe CP (GMFCS levels IV and V). Categorical variables were reported as frequencies and percentages; continuous variables as means with standard deviations (SDs) or medians with interquartile ranges (IQRs), depending on distribution. Mean z‐scores for each anthropometric index were compared between the CP and control group using linear regression, adjusted for age and sex. Prevalence of stunting, underweight, wasting and thinness was compared between groups using (chi‐square/Fisher's exact tests). A significance level of *p* < 0.05 was used throughout.

As done in the study of Polack et al. (Polack et al. 2018), the 5‐point Likert scale questions on feeding difficulties were transformed to a 0–100 ranging score (*with higher scores indicating more feeding difficulties*). Internal consistency of the questions was measured using Cronbach's alpha and inter‐item correlation. A composite feeding difficulty score was then calculated by summing the transformed item scores and categorised into tertiles representing least feeding difficulties, moderate feeding difficulties, and greatest feeding difficulties; these categories were used in subsequent analyses.

Factors associated with nutritional status within the CP group were examined using linear regression, with weight‐for‐age (WAZ) and BMI‐for‐age (BMIZ) z‐scores as continuous outcomes. These indices were selected as they are available across wider age ranges than WHZ and MUACZ, and because underweight based on WAZ has been identified as a better predictor of morbidity and mortality among children with CP than stunting or wasting, as it is not dependent on height(Brooks et al. 2011). WAZ models were fitted in children aged 24–119 months and BMIZ models in children aged 24–215 months, reflecting the ages over which each index is valid. Severe household food insecurity was defined using question 8 of the FIES—whether any household member went a whole day and night without eating because there was not enough food—as this represents the global threshold for severe food insecurity(Cafiero et al. 2017).

Two sets of estimates are reported. Age‐ and sex‐adjusted estimates were obtained by modelling each variable separately with adjustment for age in months and sex. Fully adjusted estimates were obtained from a single model including age, sex, GMFCS level, CP subtype, epilepsy, feeding difficulty score, severe household food insecurity and school attendance, with each estimate mutually adjusted for the others. Children with missing data on any included variable were excluded from the fully adjusted models, which therefore rest on smaller numbers. Previous admission for malnutrition and previous enrolment in an outpatient therapeutic programme were modelled separately, adjusted for age, sex and GMFCS level only, as contact with nutrition services may follow from poor nutritional status rather than precede it and treating these as determinants would not be appropriate. For categorical predictors with more than two levels, one category was used as the reference group. Regression coefficients (β), 95% confidence intervals (CI), and associated p‐values were reported. Statistical significance was defined as *p* < 0.05.

Use of artificial intelligence tools AI‐assisted language tools (Claude, Anthropic) were used solely for language checking and rephrasing of the manuscript. No AI tools were used for data collection, analysis, or interpretation of results. All content was critically reviewed, revised, and approved by the authors, who take full responsibility for the accuracy and integrity of the work.

### 2.6 Ethics Statement

Ethical approval for this study was obtained from the [COMREC, e.g., Malawian Medical Ethical Board] (Approval No. [P.08/22/3714]). Written informed consent was obtained from all participants. All methods were carried out in accordance with relevant guidelines and regulations.

## 3 Results

A total of 260 children with cerebral palsy (CP) and 259 age‐ and sex‐matched controls were included; see participant flow diagram (Figure 1). Median age was similar across groups (CP: 49.5 months, IQR 30–88.5; controls: 50.0 months, IQR 29.5–87.5). Sex was also equally distributed with boys comprising 56.2% of the CP group and 55.2% of the control groups (Table 1). Among children with CP, 76.2% had spastic CP, and 47.3% were classified as having severe motor impairment (GMFCS levels IV–V). Only 18.5% of children with CP were attending school, compared to 56.0% of their peers without CP (Table 2).

**Table 1:** Socio‐demographics characteristics of children with cerebral palsy and controls.

	CP (N = 260)		Controls (N = 259)	
	N	%	N	%
Age in months (median, IQR)^†^	49.50 (30.00–88.50)		50,00 (29,50–87,50)	
Age group				
24–59 months	155	59.6	155	59.8
60–119 months	67	25.8	67	25.9
≥ 120 months	38	14.6	36	13.9
Male	146	56.2	143	55.2
Attending school	48	18.5	145	56.0
Recruitment location				
Inpatient clinic	65	25.0	—	—
Outpatient clinic	179	68.8	—	—
Home visits	15	5.8	—	—
Door‐to‐door	—	—	259	100
Missing	1	0.4	0	0
Caregiver age (years) (median, range)	29 (16–67)		29 (24,00–38,00)	
Caregiver female	250	96.2	253	97.7
Caregiver relation to child				
Mother	206	79.2	219	84.6
Father	10	3.8	2	0.8
Grandmother	33	12.7	16	6.2
Other	10	3.8	15	5.8
Missing	1	0.4	7	2.7
Family size				
< 3	19	7.3	8	3.1
3	46	17.7	40	15.4
4	31	11.9	60	23.2
5	52	20	49	18.9
> 5	109	41.9	98	37.9
Missing	3	1.2	4	1.5
Caregiver Marital status				
Unmarried	5	1.9	4	1.5
Married	177	68.1	197	76.1
Divorced	66	25.4	46	17.8
Widowed	11	4.2	4	1.5
Missing	1	0.4	8	3.1
Caregiver Work status				
Unemployed/piece work	76	29.2	23	8.9
Farmer	109	41.9	173	66.8
Business	54	20.8	36	13.9
Employed	10	3.8	23	8.9
Missing	11	4.2	4	1.5
Caregiver education level				
None	52	20	32	12.4
Primary	161	62	152	58.7
Secondary	45	17.3	60	23.2
College or higher	1	0.4	10	3.9
Missing	1	0.4	5	1.9
Caregivers report affording 3 meals per day – yes	95	36.5	73	28.2

†Child's age is presented as median and interquartile range (IQR), caregiver age as median with age range, all other variables are presented as number and proportion. All percentages are calculated on children with complete recall data (251 children with CP, 246 controls). All other percentages are calculated on the full group; missing values are reported per variable.

**Table 2:** Clinical and disability characteristics of children with cerebral palsy (N = 260).

	N	%
CP subtype		
Spastic	198	76.2
Ataxic	1	0.4
Athetoid/dyskinetic	23	8.9
Hypotonic	14	5.4
Mixed	2	0.8
Missing	22	8.5
Epilepsy – yes	69	26.5
GMFCS		
Mild (I‐II)	57	21.9
Moderate (III)	59	22.7
Severe (IV‐V)	123	47.3
Missing	21	8.1
Ever admitted to hospital because of malnutrition, yes	101	39
Ever admitted to OTP programme, yes	111	42.9

*Note:* Percentages are calculated on N = 260. Age group: 24–215 months. GMFCS, Gross Motor Function Classification System. OTP refers to Outpatient Therapeutic Programme.

Caregivers of children with CP were predominantly female (96.2%), with most reportedly being the biological mother (79.2%). Caregivers in the CP group were less likely to be married (68.1% vs. 76.1%) and more likely to be unemployed or engaged in *ganyu* (casual, short‐term informal labour) (29.2% vs. 8.9%) than caregivers of controls (Table 1). Education levels were lower among caregivers of children with CP, with 20% having no formal education (vs. 12.4% in controls).

### 3.1 Anthropometric Measurements

Children with CP exhibited significantly worse nutritional status compared to their peers without CP across all anthropometric indicators (all *p* < 0.001; Figure 2, Table 3). There was no significant difference in the mean anthropometric z‐scores between boys and girls within the CP group. A large proportion of children with CP in this sample were malnourished. Underweight was observed in 81.5% of children with CP compared with 33.2% of controls, and stunting in 90.9% versus 45.6%, respectively. Thinness showed a similar pattern(39.4% vs 17.4%), with higher prevalence among children with CP than controls *p* < 0.05), with severe thinness in 23.2% versus 7.7%. Within the CP group, mean anthropometric z‐scores did not differ significantly between boys and girls (*p* < 0.05).

**Figure 2:**
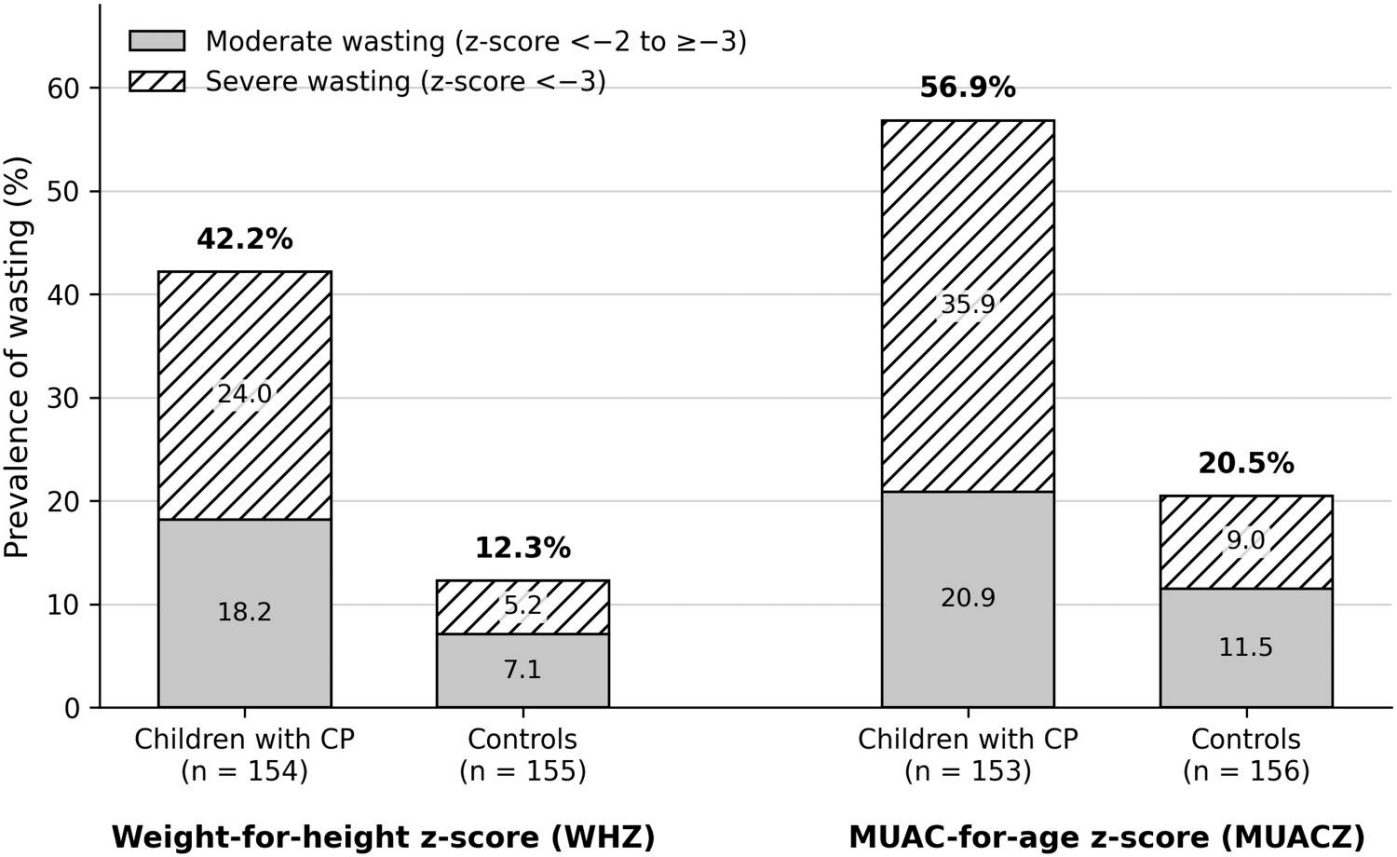
Prevalence of wasting by weight‐for‐height z‐score (WHZ) and MUAC‐for‐age z‐score (MUACZ) in children with cerebral palsy and matched controls, aged 24–59 months.

**Table 3:** Nutrition status of children with CP versus controls.

	CP (N = 260)	Controls (N = 259)	Coefficient, *p* value (95% CI)#
Weight (kg) (24–215 months)	12.31 (6.70)	16.79 (8.33)	−4.52, < 0.001 [−5.28, −3.77]
Length (cm) (24–215 months)	90.91 (18.11)	100.72 (23.23)	−9.25, < 0.001 [−11.31, −7,19]
Weight‐for‐height z‐score (mean (SD)) (24–59 months)	−1.68 (2.02)	0.02 (1.69)	−1,66, < 0.001 [−2.08, −1.25]
WHZ > 2	N = 5, 3.25%	N = 18, 11.6%	
WHZ < −2	N = 65, 42.2%	N = 19, 12.3%	
WHZ < −3	N = 37, 24.0%	N = 8, 5.2%	
Weight‐for‐age z‐score (mean (SD)) (24‐119 months)	−3.59 (1.68)	−1.33 (1.48)	−2.25, < 0.001 [−2.54, −1.97]
WAZ > 2	N = 0, 0%	N = 4, 1.8%	
WAZ < −2	N = 180, 81.5%	N = 74, 33.2%	
WAZ < −3	N = 143, 64.7%	N = 23, 10.3%	
Height‐for‐age z‐score (mean (SD)) (24‐215 months)	−4.12 (1.79)	−1.88 (1.57)	−2.24, < 0.001 [−2.54, −1.95]
HAZ > 2	N = 1, 0.39%	N = 3, 1.2%	
HAZ < −2	N = 231, 90.9%	N = 118, 45.6%	
HAZ < −3	N = 191, 75.2%	N = 60, 23.2%	
MUAC (cm age‐adjusted) (mean (SD)) (24–120 months)§	13.76 (2.69)	15.54 (2.57)	−1.81, < 0.001 [−2.19, −1.45]
Normal MUAC	N = 125, 56.8%	N = 191, 86.4%	
Moderate wasting	N = 43, 19.5%	N = 20, 9.0%	
Severe wasting	N = 52, 23.6%	N = 10, 4.5%	
MUAC‐for‐age z‐score (24–59 months)	−2.46 (1.59)	−0.97 (1.30)	−1.58, < 0,001 [−1.97, −1.19]
MUACZ < −2	N = 87, 56.9%	N = 32, 20.5%	
MUACZ < −3	N = 55, 35.9%	N = 14, 9.0%	
BMI (24–215 months)	14.15 (3.03)	15.71 (2.75)	−1.55, < 0.001 [−2.05, −1.04]
BMI‐for‐age z‐score (24–215 months)	−1.51 (2.40)	−0.29 (2.00)	−1.22, < 0.001 [−1.58, −0.85]
BAZ > 2	N = 13, 5.12%	N = 26, 10.0%	
BAZ < −2	N = 100, 39.4%	N = 45, 17.4%	
BAZ < −3	N = 59, 23.2%	N = 20, 7.7%	

*Note:* MUAC wasting categories were defined using age‐adjusted cut‐offs: for children aged 6–59 months, normal (≥ 12.5 cm), moderate (11.5–12.4 cm), and severe (< 11.5 cm); for children aged 60–120 months, normal (≥ 14.5 cm), moderate (13.5–14.4 cm), and severe (< 13.5 cm). The MUAC mean difference was estimated using linear regression adjusted for age in months.

#linear regression adjusted for age and sex.

Because the eligible age range was wide, prevalence was also examined by age band (Table S4). Differences between groups were evident in every age, but the pattern differed by indicator. Stunting among children with CP remained between 85.7% and 92.2% across bands, whereas prevalence among controls fell from 50.3% in those aged 24–59 months to 47.2% at 120 months or older; severe stunting fell from 31.0% to 8.3% in controls while remaining above 71% in children with CP. Severe thinness among children with CP rose across age bands, from 16.3% at 24–59 months to 42.9% at 120 months or older, although the difference from controls in the oldest band did not reach significance (*p* = 0.079).

#### 3.1.1 Wasting by Age Group

Using age‐specific MUAC cut‐offs, wasting was more prevalent among children with cerebral palsy than controls in both age groups (Table 4). Among children aged 2–5 years, 37.3% of children with CP were wasted compared with 9.7% of controls, and among children aged 60‐120 months, 56.7% of children with CP were wasted compared with 22.7% of controls (Fisher's exact *p* < 0.01 for both)Mean MUAC was significantly lower in children with CP in both bands, with age‐adjusted differences of 1.66 cm (24–59 months) and 1.60 cm (60–120 months), both *p* < 0.01. Among children aged 24–59 months, wasting assessed using WHZ was also substantially more common in the CP group (42.2%) than in controls (12.3%).

**Table 4:** Prevalence of wasting (with MUAC) among children with cerebral palsy and controls, aged 24–120 months (*n* = 220 CP; *n* = 220 controls).

Age group	MUAC outcome	CP (N)	Controls (N)	β (95% CI), *p*‐value†
2–5 years (24–59 months)	MUAC (cm), mean (SD)	12.91 (1.66) (N = 153)	14.57 (1.52) (N = 154)	−1.66 (−2.02 to −1.30), *p* < 0.001
	Normal MUAC, n (%)	96 (62.7)	139 (90.3)	—
	Moderate MUAC wasting, n (%)	29 (19.0)	14 (9.1)	—
	Severe MUAC wasting, n (%)	28 (18.3)	1 (0.6)	—
5–10 years (60–120 months)	MUAC (cm), mean (SD)	14.06 (2.22) (N = 67)	15.64 (1.76) (N = 66)	−1.60 (−2.25 to −0.94), *p* < 0.001
	Normal MUAC, n (%)	29 (43.3)	51 (77.3)	—
	Moderate MUAC wasting, n (%)	14 (20.9)	6 (9.1)	—
	Severe MUAC wasting, n (%)	24 (35.8)	9 (13.6)	—

†β coefficients represent age‐adjusted mean differences in MUAC estimated using linear regression models adjusted for age in months. MUAC wasting categories were defined using age‐specific cut‐offs: • 24–59 months: normal ≥ 12.5 cm; moderate 11.5–12.4 cm; severe < 11.5 cm • 60–120 months: normal ≥ 14.5 cm; moderate 13.5–14.4 cm; severe < 13.5 cm.

### 3.2 Feeding Difficulties

The feeding difficulty questionnaire was administered only to caregivers of children with CP; the findings in this section therefore apply to that group alone. In terms of psychometric properties, missing data for the eight caregiver‐reported feeding items was minimal (< 1%), and internal consistency was acceptable (Cronbach's alpha = 0.78), close to the 0.8 of the original article(Polack et al. 2018), indicating that the composite score reliably captured feeding difficulties in children with CP.

Feeding difficulties were commonly reported across multiple domains (Figure S1). Over half of caregivers of children with CP reported that their child experienced feeding problems at least ‘sometimes’ in most domains. Regarding duration of mealtimes, 87.9% responded that their child took “somewhat longer” mealtimes, meaning more than another child in the same age but less than 1 h. While 22,1% reported their child ‘always’ or ‘often’ had difficulty eating or drinking, 11,1% reported frequent coughing or choking during meals, and 33.6% reported their child was (almost) never eating enough. 39% of children were previously hospitalised due to undernutrition and 42.9% had ever participated in Outpatient Therapeutic Programme (OTP) within the group of children with CP. (Table S1). Feeding difficulties were overall moderately prevalent, with a mean overall score of 331 on a 0–800 scale (8 questions with a total of 100 per question), where lower values indicate lesser feeding challenges.

Feeding challenges were significantly higher among children who had ever been admitted to hospital (*p* = 0.0009) and those ever enroled in OTP (*p* = 0.0023), indicating that more severe feeding difficulties were associated with nutritional treatment needs. There were no significant associations between history of hospital or OTP admission and mealtime duration, nor caregiver‐reported growth status.

### 3.3 Feeding Practises

64.7% of children in the CP group were feeding‐dependent compared to 14.5% in the control group, with a statistically significant difference (x^2 = 136.217, *p* < 0.001). This difference remained statistically significant when stratified for age (children 2–4 years = x^2 81.002, *p* < 0.001 and children aged 5 years or older= x^2 65.339, *p* < 0.001). GMFCS levels of III‐V were significantly associated with being feeding‐dependent (45.906, *p* < 0.001) (Table S2).

#### 3.3.1 Household Food Security and Dietary Diversity

Food insecurity was highly prevalent in both groups, affecting 63.5% of the CP group and 71.1% of the control group over the preceding 4 weeks. The difference was not statistically significant (*p* = 0.068). However, severe food insecurity was significantly more common in the CP group (13.5%) than in the non‐CP group (7.5%), (*x* ^2^ = 4.559, *p* = 0.033).

Dietary intake, assessed by caregiver report of food groups consumed on a typical day, was narrow in both groups (Table S5). Cereals and cereal products were consumed by 98.4% of children with CP and 95.1% of controls, whereas meats and poultry (4.8% and 5.3%) and eggs (6.4% and 12.2%) were rarely consumed in either group.

### 3.4 Factors Associated With Malnutrition

To explore factors associated with nutritional status among children with CP, linear regression analysis was performed for WAZ and BMIZ as outcome variables (Table 4). Children with CP were at increased risk of being underweight or thin as they grew older, with significant negative associations between age and both WAZ and BMIZ (*β* = −0.01 for both, *p* < 0.001). Sex of the child and presence of epilepsy were not significantly associated with nutritional status. School attendance was associated with higher WAZ (*p* = 0.001).

Children classified with moderate and severe gross motor function impairment (GMFCS) had significantly lower WAZ scores compared to those with mild impairment. Severe GMFCS was also significantly associated with lower BMIZ scores. Children with spastic CP had significantly worse WAZ and BMIZ scores compared to those with other subtypes (*p* = 0.005 and *p* < 0.001, respectively).

Caregiver characteristics—including age, education level, employment and family size—did not show associations with underweight status. However, children of caregivers who were married had significantly higher WAZ and BMIZ scores compared to those with unmarried, widowed, or divorced caregivers (*p* = 0.02 and *p* = 0.05, respectively) (Table S3).

Feeding difficulties were strongly associated with poorer nutritional status. Children whose caregivers reported frequent problems with eating or drinking, coughing or choking, needing help with feeding, caregiver unhappiness, and caregiver worries about feeding had significantly lower WAZ and BMIZ scores. Notably, children who required help with feeding often or always had substantially lower WAZ (*β* = −1.54, *p* < 0.001) and BMIZ (*β* = −1.53, *p* < 0.001) compared with those who rarely or never required assistance. Meal duration was also significantly associated with nutritional status; children whose meals took longer than those of their peers had lower WAZ (*β* = −1.06, *p* = 0.01) and BMIZ (*β* = −1.11, *p* = 0.02) (Table S4, Appendix 2). Across feeding‐difficulty tertiles, progressively lower WAZ and BMIZ were observed among children with moderate and severe feeding difficulties compared with those with the least difficulties.

A history of hospital admission for malnutrition or enrolment in an OTP was significantly associated with worse nutritional status across both indicators (*p* < 0.001 for all comparisons). Lastly, caregivers reporting with severe food insecurity (“mother reported going whole day and night without food in past 2 weeks”) had children with significantly lower BMIZ scores (*β* = −1.03, *p* = 0.002), although this association did not persist after adjustment for other factors (Table 5).

**Table 5:** Factors associated with weight‐for‐age and BMI‐for‐age z‐scores among children with cerebral palsy.

	Weight‐for‐age z‐score		BMI‐for‐age z‐score	
	Age‐ and sex‐adjusted	Fully adjusted	Age‐ and sex‐adjusted	Fully adjusted
	*β* (95% CI); *p*	*β* (95% CI); *p*	*β* (95% CI); *p*	*β* (95% CI); *p*
Child and household characteristics				
Age (per month)	−0.02 (−0.02, −0.01); < 0.001	−0.02 (−0.03, −0.01); < 0.001	−0.01 (−0.02, −0.01); < 0.001	−0.01 (−0.02, −0.01); < 0.001
Female sex	0.14 (−0.30, 0.57); 0.531	−0.01 (−0.49, 0.46); 0.954	−0.23 (−0.81, 0.35); 0.442	−0.07 (−0.76, 0.62); 0.840
GMFCS III (ref. I–II)	0.14 (−0.39, 0.66); 0.607	−0.94 (−1.67, −0.21); 0.012	0.19 (−0.52, 0.90); 0.591	−1.00 (−2.05, 0.05); 0.062
GMFCS IV–V (ref. I–II)	−0.93 (−1.36, −0.49); < 0.001	−1.54 (−2.19, −0.89); < 0.001	−0.92 (−1.51, −0.32); 0.003	−1.71 (−2.65, −0.77); < 0.001
Spastic subtype (ref. other)	−0.82 (−1.45, −0.18); 0.012	−0.71 (−1.41, −0.01); 0.046	−1.37 (−2.19, −0.54); 0.001	−1.20 (−2.19, −0.21); 0.018
Epilepsy	0.16 (−0.34, 0.66); 0.535	0.27 (−0.25, 0.78); 0.306	−0.14 (−0.80, 0.53); 0.686	0.13 (−0.61, 0.88); 0.722
Feeding difficulty score (per point)	−0.03 (−0.05, −0.01); 0.006	−0.03 (−0.06, −0.01); 0.005	−0.05 (−0.07, −0.02); < 0.001	−0.04 (−0.07, −0.01); 0.016
Severe food insecurity	−0.10 (−0.80, 0.60); 0.774	−0.26 (−0.96, 0.43); 0.451	0.06 (−0.85, 0.98); 0.890	0.01 (−0.96, 0.98); 0.983
Attending school	1.20 (0.54, 1.87); < 0.001	0.92 (0.16, 1.67); 0.018	0.32 (−0.50, 1.13); 0.442	−0.50 (−1.52, 0.52); 0.333
Prior contact with nutrition services				
Previously admitted for malnutrition	−1.46 (−1.86, −1.06); < 0.001	−0.47 (−1.02, 0.09); 0.097	−1.49 (−2.06, −0.93); < 0.001	−1.20 (−2.07, −0.33); 0.007
Previously enroled in OTP	−1.62 (−2.00, −1.24); < 0.001	−1.19 (−1.74, −0.65); < 0.001	−1.25 (−1.82, −0.69); < 0.001	−0.27 (−1.12, 0.58); 0.533

*Note:* Linear regression. Age‐ and sex‐adjusted estimates were obtained by modelling each variable separately with adjustment for age in months and sex. Fully adjusted estimates are mutually adjusted for all other variables within the same section of the table. Weight‐for‐age models were fitted in children aged 24–119 months and BMI‐for‐age models in children aged 24–215 months, reflecting the ages over which each index is applicable. Fully adjusted models: *n* = 157 and 185 for child and household characteristics, and *n* = 208 and 243 for prior service contact; the smaller numbers reflect listwise exclusion of children with missing data on any included variable. Prior contact with nutrition services is presented separately, adjusted for age, sex and GMFCS level only, because such contact may follow from poor nutritional status rather than precede it. Higher feeding difficulty scores indicate more reported difficulties.

Abbreviations: GMFCS, Gross Motor Function Classification System; OTP, outpatient therapeutic programme.

## 4 Discussion

This study demonstrates an exceptionally high burden of malnutrition among children and adolescents with cerebral palsy (CP) in rural Malawi. Across all anthropometric indicators, children with CP had significantly worse nutritional status than age‐ and sex‐matched controls from the same area (without CP). Severe wasting was already evident in early childhood among children with CP, and appeared to be even more pronounced at older ages.

When compared with Mangochi and national nutrition data, where wasting affects 1.2% of children in Mangochi and 2.0% nationally, and stunting approximately 40.6% and 37.6%(National Statistical Office 2025)—both groups exhibited substantially worse outcomes, with the CP group showing the greatest disadvantage. National estimates cover only children under five, so this comparison should be read with caution; restricting our data to that age group gives similar or higher prevalence in both groups (stunting 92.2% and 50.6%), so the difference is not overstated. Here, differences in the timing and method of data collection need to be taken into account, as our enrolment spanned the lean season, when household food insecurity is greatest. Stunting was more than twice as common among controls (45.6%) compared to the national average, and more than twice as high again in children with CP (90.9%), placing the CP group at three times the regional SSA rate of 30.7% (World Bank group 2023). Wasting in the CP group (42.2%) was more than 20 times the district prevalence, while even controls (12.2%) showed a sixfold higher wasting rate than the general Mangochi population. Underweight affected nearly two and a half times as many children with CP as controls.

These elevated rates reflect both the broader and structural context and the additional vulnerabilities specific to CP. Because controls were recruited from the same communities and individually matched, this distinction can be drawn directly: the disadvantage associated with CP is incremental to an already high baseline rather than a reflection of it. At the population level, household food insecurity is pervasive, affecting 63.5% of CP households and 71.1% of control households—consistent with patterns described across SSA, where widespread poverty, limited market access, seasonal instability and structural inequalities drive household‐ level food insecurity despite national food availability(Lungu et al. n.d.). The pattern differs by severity. Overall food insecurity was not more prevalent among CP households, yet severe food insecurity was significantly more common (13.5% vs 7.5%). Severe food insecurity may therefore compound the child‐level feeding difficulties associated with the nutritional deficit rather than explain it: control children were more often food insecure overall, yet substantially better nourished. Dietary intake was narrow in both groups, with animal‐source foods rarely consumed, indicating that the nutritional deficit in children with CP is unlikely to be explained by household dietary quality alone. Within this environment, children with CP face additional risks related to feeding difficulties, prolonged mealtimes, caregiver fatigue and the absence of disability‐specific nutrition guidance or rehabilitation support(Engl et al. 2022; L. Jahan et al. 2023; Polack et al. 2018). Caregivers of children with CP were also three times more likely to report unemployment or piece work (29.2% vs 8.9%) and less likely to be farming (41.9% vs 66.8%), which requires sustained absence from home and is difficult to combine with assisted feeding at every meal—suggesting reduced employment is partly a consequence of caregiving rather than a cause of it (Chiluba and Moyo 2017; Patel et al. 2017; van der Meijden, Wedda, et al. 2026). Offering a plausible route to the more severe food insecurity noted above. Stunting was established by early childhood in both groups, but declined with age only among controls, while thinness became more common with age in the CP group. Linear growth deficit therefore appears to be set early, while deficits in weight relative to height accumulate, consistent with reports that older age predicts undernutrition among children with CP in LMIC settings (Jahan et al. 2024; Kakooza‐Mwesinge et al. 2017). Two mechanisms are plausible. Energy requirements may rise with body size without a corresponding increase in intake capacity. Caregiver capacity may also change over time, as the physical demands of assisted feeding increase with the child's weight while the caregiver ages and other household responsibilities accumulate (van der Meijden, Wedda, et al. 2026). As these data are cross‐sectional, they describe differences between age groups rather than change within individuals.

Our findings align with, but in several respects surpass, estimates from other sub‐Saharan African CP studies, which report 50%–85% for underweight or “any malnutrition” and 38%–74% stunting (Adamu et al. 2018; I. Jahan et al. 2024; Makhoul et al. 2025; Namaganda et al. 2023; Polack et al. 2018). The results are consistent with meta‐analytic evidence showing that children with disabilities in LMICs are almost three times more likely to be underweight and twice as likely to be stunted or wasted (Hume‐Nixon and Kuper 2018).

Motor severity was strongly associated with nutritional status, and this association appeared to operate through feeding. Children with more severe motor impairment were significantly more likely to be feeding‐dependent, and feeding dependency was common in this population (64.7% vs 14.5% in controls), higher than described in other international papers (Nyman et al. 2025; Schepers et al. 2022). Feeding difficulties were widespread and spanned multiple domains: nearly 90% of caregivers reported prolonged mealtimes, 11.1% reported frequent coughing or choking, and one‐third indicated that their child (almost) never ate enough. Higher feeding difficulty scores were significantly associated with poorer WAZ and BMIZ outcomes, consistent with the central role of oropharyngeal dysfunction, prolonged mealtimes, and caregiver burden in limiting dietary intake (Arvedson 2013; L. Jahan et al. 2023). For a child who cannot self‐feed, adequate intake depends not on food being available but on a caregiver having the time and technique to deliver it at every meal, which may explain why household food insecurity and dietary intake did not differ between groups while nutritional status did(L. Jahan et al. 2023; Samuel et al. 2023). As these data are cross‐sectional, the direction of this relationship cannot be established; undernutrition may itself worsen oral‐motor function, so the association is likely to be bidirectional. Although only 22.1% of caregivers reported frequent eating or drinking difficulties, this likely reflects under‐recognition or normalisation of feeding problems in settings with limited disability‐specific education. Caregivers may perceive difficulties as typical for CP or focus on other visible health issues (Samuel et al. 2023; Taylor et al. 2022). Relying solely on caregiver report may therefore underestimate the true burden of feeding difficulties: a structured observational assessment tool, such as the Eating and Drinking Ability Classification System (EDACS) could provide a more objective basis for identifying children at nutritional risk (Sellers et al. 2014), though validation in SSA remains needed before implementation.

Beyond assessment, addressing these feeding challenges will require targeted, context‐appropriate intervention. A scoping review of nutrition interventions for children with CP in LMIC found that dietary modification—alone or as part of a broader approach—had better outcomes than behavioural feeding skills training alone, and that community‐based caregiver training incorporating positioning, responsive feeding, and dietary modification has shown promise where access to institutional care is limited(I. Jahan et al. 2022). Routine clinic visits represent a practical entry point for structural feeding assessment and practical caregiver education.

Clinical vulnerability further compounded the nutritional risk: 39% of children had been previously hospitalised for malnutrition, and 42.9% had been enrolled in an OTP, both strongly associated with poorer anthropometric outcomes. Indicating persistent nutritional vulnerability despite therapeutic intervention, and likely underrepresenting the true burden as children who did not survive will not have been captured. Child characteristics—including age, GMFCS severity, and spastic CP subtype—showed strong associations with poorer nutritional outcomes, consistent with more severe motor impairment being associated with reduced s oral intake and reduced feeding autonomy (I. Jahan et al. 2024; Namaganda et al. 2023). School attendance was associated with higher WAZ, but only 18.5% of children with CP attended school, and attendance likely reflects milder impairment and better function—a marker of functional capacity rather than a protective factor in itself. These factors could be used to empower OTP services to identify children with CP at highest risk of failed nutritional recovery. Investment in additional training of health workers in disability‐specific nutritional management is needed to improve post‐discharge outcomes for this vulnerable group. This is particularly urgent given that children with CP have been shown to have almost sixfold higher post‐discharge mortality and are nearly three times more likely to be readmitted compared to children without CP(Bwakura‐Dangarembizi et al. n.d., 2021; Kerac et al. 2014).

These findings have clear implications for policy and practice. Disability‐specific nutrition guidelines are urgently needed in Malawi, as children with CP face feeding and growth challenges that fall outside the scope of standard nutrition protocols(Engl et al. 2022). Second, existing nutrition programmes—including OTP and growth monitoring—should be equipped to specifically identify and refer children with CP, with training for health workers on disability‐specific feeding support, including positioning, texture modification, and assisted feeding techniques. Third, caregiver education on recognising and addressing feeding problems should be integrated in rehabilitation and community health programmes, equipping caregivers with the practical skills needed to support their child's nutritional needs at home. Malawi's national commitment to reduce stunting prevalence to 33% by 2030 will require explicit inclusion of children with disabilities, who are currently invisible in national nutrition data and policy frameworks (Global Nutrition report 2025). Children with CP are not a marginal category within the malnourished population but a distinct group in whom undernutrition is near‐universal, severe, and driven by mechanisms that standard nutrition protocols do not address.

### 4.1 Strengths and Limitations

Key strengths of this study include the use of an age‐ and sex‐matched control group, which enabled clear comparison of nutritional risk between children with and without CP. Comprehensive assessment using multiple anthropometric indicators and standardised GMFCS classification strengthened measurement accuracy and interpretation. The inclusion of a caregiver‐reported feeding questionnaire also provided valuable insight into mealtime challenges experienced.

This study has several limitations. Cases and controls were not sampled through identical pathways: children with CP were recruited in three places, whereas controls were recruited door‐to‐door. Because three‐quarters of children with CP were enroled through routine care or home visits rather than during acute admission, differences in acute illness are unlikely to explain the deficits observed. However, children recruited through the clinic were already engaged with multidisciplinary care; children with CP outside the health system are under‐represented, and our estimates should be regarded as conservative. Anthropometric measurement is difficult in children with severe motor impairment, for whom accurate positioning is challenging. Where deformity precluded direct measurement, knee height and the Stevenson equation were used; these carry their own prediction error, and residual error in height‐derived indices is likely. Nutritional status was assessed against WHO growth standards, which were not developed for children with disabilities, so absolute prevalence in the CP group may overstate nutritional deficit, particularly at the severe end of the motor spectrum. CP‐specific references were not used, as these describe observed rather than optimal growth in populations with their own unmet nutritional needs and may normalise clinically significant deficits. Both groups were assessed using identical standards and instruments; the differences between groups are unlikely to be attributable to the choice of reference. Several determinants were not captured. Recent morbidity was not assessed, and dietary intake was recorded only as food groups consumed, which does not permit assessment of dietary adequacy. We measured caregiver‐reported feeding difficulty but not caregiver adaptation, which is likely to mediate the relationship between feeding difficulty and nutritional status. Mealtime duration and caregiver‐reported growth were collected only in the CP group, limiting direct comparison. Finally, some analytical constraints apply. The sample was weighted towards younger children (median 49.5 months; 59.6% under 60 months), so estimates for the oldest age band rest on smaller numbers and were less precise. The single‐district setting may limit generalisability, although the findings align with regional evidence.

## 5 Conclusion

This study demonstrates an exceptionally high burden of malnutrition among children with cerebral palsy (CP) in rural Malawi, with underweight, stunting and wasting rates exceeding national levels, regional averages and those of matched peers. The high prevalence of prior outpatient nutrition care suggests that many children with cerebral palsy have accessed nutrition services, yet substantial levels of undernutrition persist. Severe motor impairment, widespread feeding difficulties, recurrent illness and household food insecurity were key contributors. These findings underscore the urgent importance of disability‐inclusive nutrition services, improved caregiver support and practical feeding guidance. Strengthening integrated nutrition and rehabilitation care is essential to improve health, growth and developmental outcomes for this highly vulnerable population.

## Author Contributions

BvdM and NL conceptualised, designed, collected and analysed the data of the study and drafted the manuscript. AC, LC and MM were part of reviewing of the manuscript. WV supervised the analysis and drafting of the manuscript. All authors read and approved the final manuscript.

## Funding

The authors have nothing to report.

## Conflicts of Interest

The authors declare no conflicts of interest.

## Supporting information


Supporting File

## Data Availability

The data that support the findings of this study are available on request from the corresponding author. The data are not publicly available due to privacy or ethical restrictions. The datasets used and/or analysed during the current study are available from the corresponding author on reasonable request.
