# Supplementary material for: Prevalence of Malnutrition and Contributing Factors in Children With Cerebral Palsy in Malawi: A Case‐Control Study
Source: Matern Child Nutr. 2026 Oct 29;22(4):e70260. doi: 10.1111/mcn.70260 (PMC13717596; doi:10.1111/mcn.70260)
Supplement: Supplementary file 1 [file MCN-22-e70260-s001.docx]

# Appendix 1. Questionnaire

1. Baseline demographics kind

| Name |  | | | | | |
| --- | --- | --- | --- | --- | --- | --- |
| Village / TA |  | | | | | |
| Age |  | | | | | |
| Sex | M | | | F | | |
| CP severity (GMFCS) | 1 | 2 | 3 | | 4 | 5 |
| School | yes | | | no | | |
| If yes which class |  | | | | | |

2. Baseline demographic caregiver

| Name |  | | | | | | | | | | |
| --- | --- | --- | --- | --- | --- | --- | --- | --- | --- | --- | --- |
| Village / TA |  | | | | | | | | | | |
| Age |  | | | | | | | | | | |
| Sex | M | | | | F | | | | | | |
| Relationship to child | Mother | | Father | | Grandparent | | | Other specify: | | | |
| Family size | <3 | | 3 | | 4 | | 5 | | | | >5 |
| Relationship status | Married | Widowed | | | Divorced | | | | | Single | |
| Work status | Unemployed | | Employed | | Farmer | | Business | | | | Other specify |
| Able to effort 3 meals a day | yes | | | | no | | | | | | |
| Highest level op education | No education | | | Primary | | Secondary | | | College or higher | | |

1. Feeding Questions (derived from Polack et al)

|  | 0 | 1 | 2 | 3 | 4 |
| --- | --- | --- | --- | --- | --- |
| Does your child have any problems with eating and drinking? | Never | Almost Never | Sometimes | Often | Always |
| Does your child eat the same food as other children the same age? | Never | Almost Never | Sometimes | Often | Always |
| Does your child cough or choke while eating or drinking? | Never | Almost Never | Sometimes | Often | Always |
| Does your child feed himself/herself? | Never | Almost Never | Sometimes | Often | Always |
| Does your child need help with feeding? | Never | Almost Never | Sometimes | Often | Always |
| Is your child unhappy during mealtimes | Never | Almost Never | Sometimes | Often | Always |
| Do you think your child is eating enough? | Never | Almost Never | Sometimes | Often | Always |
| Do these problems worry you? | Never | Almost Never | Sometimes | Often | Always |
| How long does it take for your child to have a meal? |  | faster than other child of the same age | The same as other child of the same age | Somewhat longer than other child of the same age | (more than 1 hour) much longer than other child of the same age |
| Has your child ever been admitted to the hospital for malnutrition or eating/drinking difficulties? * | No | Yes |  | | |
| Has your child ever been included in the OTP program?* | No | Yes |  | | |
| Is your child growing and putting on weight like other children? | No | Yes |  | | |
| If not, what do you believe is the reason your child is not growing and putting on weight like other children? |  | | | | |

*Added questions outside Polack et a. questionnaire

**Child Dietary Diversity questionnaire**

**Child Dietary Diversity**

**What does the child eat on a typical day?**

- *Ask this as an open question and select all that the caregiver mentions.*
- *Do not present the caregiver with this list.*
- *You may prompt the caregiver with open questions, e.g. What does your child usually eat for breakfast*

❑ **Milk and Milk Products**: Fresh/fermented milk, cheese, yogurt, or other milk products

❑ **Breast milk**

❑ **Cereals and Cereal Products**: Maize, rice, pasta, porridge, bread, biscuits, millet, sorghum, wheat, locally available grains

❑ **Fish and Sea Foods**: fresh or dried fish or shellfish

❑ **Roots and Tubers**: potatoes, sweet potatoes, yams, cassava, or foods made from roots or wild roots and tubers

❑ **Vegetables**: Cabbages, carrots, spinach, and any other locally available vegetables including wild vegetables

❑ **Fruits**: Oranges, bananas, mangoes, avocados, apples, grapes etc

❑ **Meats and Poultry**: Camel, beef, lamb, goat, rabbit, wild game, chicken or other birds, liver, kidney, heart or other organ meats or blood-based foods

❑ **Eggs**: Hen or other bird eggs

❑ **Pulses / Legumes / Nuts and Seeds**: Beans, peas, lentils, nuts, seeds or foods made from these

❑ **Fats and Oils**: Oil, fats, ghee, margarine or butter added to food or used for cooking

❑ **Sugars / Honey and Commercial Juices**: Sugar in tea, honey, sweetened soda, juices, chocolates, sweets or candies

❑ **Miscellaneous**: Spices, unsweetened beverages

# Appendix 2. Supplemental data

**Figure S1**. Caregiver-reported feeding difficulties

Caregiver-reported difficulties with child's feeding in percentage (%). Figure based on the figure presented in the Polack et al. study

Feeding difficulties

|  | Never | Almost never | Sometimes | Often | Always |
| --- | --- | --- | --- | --- | --- |
| problems eating drinking | 56.4 | 2.3 | 18.9 | 1.5 | 20.8 |
| eats same as peers | 14.8 | 1.2 | 18.9 | 16.2 | 49 |
| cough or choke | 52.5 | 6.6 | 29.7 | 2.7 | 8.5 |
| able to feed themselves | 60.2 | 1.9 | 5.8 | 4.3 | 27.8 |
| need help feeding | 23.6 | 3.4 | 7.4 | 3.9 | 61.8 |
| caregiver unhappy | 75.1 | 1.9 | 13.6 | 0.8 | 8.6 |
| eating enough | 29.1 | 3.5 | 38.4 | 10.8 | 18.2 |
| caregiver worries | 39.7 | 4.6 | 32.4 | 6.5 | 16.6 |

**Table S1**. Feeding difficulties for children with CP

|  | **Q1. problems eating drinking** | **Q2. eats same as peers** | **Q3. cough or choke** | **Q4. able to feed themselves** | **Q5. need help feeding** | **Q6. caregiver unhappy** | **Q7. eating enough** | **Q8. caregiver worries** |
| --- | --- | --- | --- | --- | --- | --- | --- | --- |
| Never | 149(56,9) | 41(15,6) | 139(53,1) | 159(60,7) | 64 (24,4) | 198(75,6) | 79(30,2) | 106(40,5) |
| Almost never | 6(2,3) | 3(1,1) | 17(6,5) | 5(1,9) | 9(3,4) | 5(1,9) | 9(3,4) | 12(4,6) |
| Sometimes | 49(18,7) | 49(18,7) | 77(29,4) | 15(5,7) | 19(7,3) | 35(13,4) | 99(37,8) | 84(32,1) |
| Often | 4(1,5) | 42(16,0) | 7(2,7) | 11(4,2) | 10(3,8) | 2(0,8) | 28(10,7) | 12(6,5) |
| Always | 54(20,6) | 127(48,5) | 22(8,4) | 72(27,5) | 158(60,3) | 22(8,4) | 47(17,9) | 106(16,4) |

**Describe (N total is 262) N=(%):**

*For further analysis of Cronbach alpha and dividing in tertiles, Q2,Q4 and Q7 recoded for.

|  | Faster than peers n= (%) | Same as peers n= (%) | Somewhat longer than peers n= (%) | More than >1 hr (n= (%) |
| --- | --- | --- | --- | --- |
| **Q9. Mealtime duration** | 4, (1,6) | 21 (8,1) | 228 (88) | 5 (1,9) |

|  | Yes n= (%) | No n= (%) |
| --- | --- | --- |
| **Q10. Growing like others** | 44 (17) | 214 (83) |

**Table S2**. Feeding dependency. Chi Square Test of Independence conducted between feeding dependent and feeding independent. Feeding dependent is classified as fed by an adult, all other options are classified as feeding independent

**Feeding practices**

|  | **CP group N(%)** | **Non-CP group N(%)** | **p-value (x^2 value)** |
| --- | --- | --- | --- |
| **Feeding dependent** | 154 | 35 | **<0.001** (136.217) |
| Fed by an adult | 154 (64.7) | 35 (14.5) |  |
| **Feeding independent** | 74 | 205 |  |
| Child feeds self (supervision parent) | 14 (5.9) | 22 (9.1) |  |
| Child feeds self (supervision by child) | 2 (0.8) | 0 |  |
| Feeds self. unsupervised | 57 (23.9) | 174 (72.2) |  |
| Fed from common plate | 1 (0.4) | 9 (3.7) |  |
| Exclusively breastfed | 0 (0) | 0 (0) |  |
| Unknown | 10 (4.2) | 1 (0.4) |  |
| Missing | 4 | 1 |  |
| Total | 242 | 242 |  |
|  |  |  |  |
|  | CP group N(%) | Non-CP group(%) | p-value (x^2 value) |
|  |  |  |  |
| **< 5 years** |  |  | **<0.001 (81.002)** |
| Feeding dependent | 105 (77.8) | 34 (23.8) |  |
| Feeding independent | 30 (22.2) | 109 (76.2) |  |
| Missing | 9 | 1 |  |
| **> 5 years** |  |  | **<0.001 (65.339)** |
| Feeding dependent | 49 (52.7) | 1 (1.0) |  |
| Feeding independent | 44 (47.3) | 96 (99) |  |
| Missing | 5 | 1 |  |
| Total | 242 | 242 |  |
|  | **GMFCS level I-II** | **GMFCS level III-V** | **<0.001 (45.906)** |
| Feeding dependent | 15 | 130 |  |
| Feeding independent | 38 | 35 |  |

*.*

**Table S3.** Association between WAZ and BMIZ and socio-demographic, clinical, and feeding variables among children with CP

|  | Weight-for-age Z-score | | BMI-for-age Z-score | |
| --- | --- | --- | --- | --- |
|  | Mean [SD] | Coefficient, (95% CI)  p-value | Mean [SD] | Coefficient (β), (95% CI)  p-value |
| **Child characteristics** | | | | |
| Attending school child |  |  |  |  |
| No | -3,67 [1,65] | Reference | -1,45 [2,33] | Reference |
| Yes | -3,02 [1,82] | 1,11  [0,43 , 1,78]  P=0,001 | -1,79 [2,67] | 0,31  [-0,50, 1,13]  P=0,45 |
| **Caregiver social demographics** | | | | |
| Family size* |  |  |  |  |
| <3 or equal 3 | -3,55 [1,75] | Reference | -1,65 [2,23] | Reference |
| 4 or 5 | -3,48 [1,54] | 0,27  [-0,31, 0,85]  P=0,36 | -0,80 [2,45] | 1,14  [0,40, 1,88]  P=0,003 |
| >5 | -3,74 [1,75] | 0,01  [-0,53, 0,56]  P=0,96 | -1,98 [2,34] | 0,08  [-0,62, 0,77]  P=0,83 |
| Caregiver Marital status |  |  |  |  |
| Unmarried/ widowed/ divorced | -4,05 [1,49] | Reference | -2,08 [2,18] | Reference |
| Married | -3,43 [1,72] | 0,57  [0,10, 1,05]  P=0,02 | -1,35 [2,29] | 0,60  [0,01, 1,19]  P=0,05 |
| Caregiver Work status |  |  |  |  |
| Unemployed / piece work | -3,68 [1,50] | Reference | -1,34 [1,88] | Reference |
| Farmer | -3,66 [1,83] | -0,13  [-0,86, 0,60]  P=0,72 | -1,73 [2,51] | -0,78  [-1,66, 0,09]  P=0,08 |
| Business/employed | -3,44 [1,70] | -0,21  [-0,58, 1,00]  P=0,61 | -1,27 [2,29] | -0,09  [-1,02, 0,84]  P=0,86 |
| Caregiver education level | Also checked if it differs for severity |  |  |  |
| None | -3,85 [1,75] | Reference | -1,52 [2,46] | Reference |
| Primary | -3,57 [1,69] | -0,27  [-0,28, 0,82]  P=0,33 | -1,63 [2,26] | -0,03  [-0,73, 0,68]  P=0,94 |
| Secondary or higher | -3,40 [1,58] | -0,47  [-0,24, 1,18]  P=0,19 | -1,35 [2,13] | -0,20  [-0,71, 1,11]  P=0,66 |
| Caregivers report affording 3 meals per day |  |  |  |  |
| No | -3.67 (1.56) | Reference | -1.59 (2.22) | Reference |
| Yes | -3.49 (1.86) | 0.18  (-0.27, 0.63)  P=0.43 | -1.49 (2.38) | -0.01  (-0.58, 0.56)  P=0.97 |
| **Feeding difficulties questions** |  |  |  |  |
| Problems eating drinking |  |  |  |  |
| (almost) never (0,1) | -3.27 (1.64) | Reference | -0.98 (2.32) | Reference |
| Sometimes (2) | -4.13 (1.63) | -1.06  (-1.60, -0.52)  P<0.001 | -2.11 (2.25) | -1.40  (-2.14, -0.66)  P<0.001 |
| Often/always (3,4) | -4.03 (1.57) | -0.80  (-1.31, -0.28)  P=0.003 | -2.38 (2.39) | -1.45  (-2.13, -0.76)  P<0.001 |
| Cough / choking |  |  |  |  |
| (almost) never (0,1) | -3.34 (1.76) | Reference | -1.13 (2.48) | Reference |
| Sometimes (2) | -3.98 (1.52) | -0.58  (-1.06, -0.10)  P=0.02 | -1.87 (2.18) | -0.65  (-1.29, -0.01)  P=0,048 |
| Often/always (3,4) | -4.11 (1.18) | -0.85  (-1.55, -0.15)  P=0.02 | -2.61 (2.06) | -1.55  (-2.46, -0.63)  P=0.001 |
| Need help feeding |  |  |  |  |
| (almost) never (0,1) | -2.79 (1.62) | Reference | -1.13 (2.51) | Reference |
| Sometimes (2) | -2.91 (1.55) | -0.76  (-1.64, 0.13)  P=0.09 | -0.28 (3.59) | 0.04  (-1.14, 1.22)  P=0.95 |
| Often/always (3,4) | -3.91 (1.60) | -1.54  (-2.05, -1.01)  P<0.001 | -1.81 (2.13) | -1.53  (-2.23, -0.82)  P<0.001 |
| Caregiver unhappy |  |  |  |  |
| (almost) never (0,1) | -3.30 (1.58) | Reference | -1.02 (2.21) | Reference |
| Sometimes (2) | -4.72 (1.52) | -1.39  (-1.98, -0.78)  P<0.001 | -2.94 (2.11) | -1.90  (-2.71, -1.09)  P<0.001 |
| Often/always (3,4) | -4.59 (1.57) | -1.33  -2.09, -0.57  P=0.001 | -3.35 (2.62) | -2.15  (-3.09, -1.20)  P<0.001 |
| Caregiver worries |  |  |  |  |
| (almost) never (0,1) | -3.11 (1.71) | Reference | -1.27 (2.32) | Reference |
| Sometimes (2) | -3.86 (1.52) | -0.66  (-1.14, -0.18)  P=0,007 | -1.27 (2.45) | 0.05  (-0.62, 0.71)  P=0.89 |
| Often/always (3,4) | -4.23 (1.51) | -1.07  (-1,61, -0.54)  P<0.001 | -2.32 (2.33) | -0.98  (-1.72, -0.25)  P=0.01 |
| Duration of mealtime |  |  |  |  |
| Same or faster as peers (group 1 +2) | -2,55 [1.46] | Reference | -0,63 [2,17] | Reference |
| Longer (3+4) | -3,71 [1,66] | -1,06  [-1,83, -0,30]  P=0,01 | -1,65 [2,27] | -1,11  [-2,04, -0,18]  P=0,02 |

| **Table S4. Prevalence of undernutrition in children with cerebral palsy and matched controls, by age group** | | | | | | | | | |
| --- | --- | --- | --- | --- | --- | --- | --- | --- | --- |
|  |  |  |  |  |  |  |  |  |  |
| **Indicator** | **24–59 months** | | | **60–119 months** | | | **≥120 months** | | |
|  | **CP** | **Control** | **p** | **CP** | **Control** | **p** | **CP** | **Control** | **p** |
| Stunting (HAZ <−2) | 141/153 (92.2) | 78/155 (50.3) | <0.001 | 60/66 (90.9) | 22/67 (32.8) | <0.001 | 30/35 (85.7) | 17/36 (47.2) | <0.001 |
| Severe stunting (HAZ <−3) | 118/153 (77.1) | 48/155 (31.0) | <0.001 | 48/66 (72.7) | 9/67 (13.4) | <0.001 | 25/35 (71.4) | 3/36 (8.3) | <0.001 |
| Thinness (BAZ <−2) | 48/153 (31.4) | 20/155 (12.9) | <0.001 | 33/66 (50.0) | 13/67 (19.4) | <0.001 | 19/35 (54.3) | 12/36 (33.3) | 0.096 |
| Severe thinness (BAZ <−3) | 25/153 (16.3) | 8/155 (5.2) | 0.002 | 19/66 (28.8) | 4/67 (6.0) | <0.001 | 15/35 (42.9) | 8/36 (22.2) | 0.079 |
| Underweight (WAZ <−2) | 123/153 (80.4) | 48/155 (31.0) | <0.001 | 55/65 (84.6) | 26/66 (39.4) | <0.001 | – | – | – |
| Severe underweight (WAZ <−3) | 95/153 (62.1) | 14/155 (9.0) | <0.001 | 46/65 (70.8) | 9/66 (13.6) | <0.001 | – | – | – |
| Wasting (WHZ <−2) | 65/153 (42.5) | 19/153 (12.4) | <0.001 | – | – | – | – | – | – |
| Severe wasting (WHZ <−3) | 38/153 (24.8) | 8/153 (5.2) | <0.001 | – | – | – | – | – | – |
| Wasting (MUACZ <−2) | 87/153 (56.9) | 31/153 (20.3) | <0.001 | – | – | – | – | – | – |
| Severe wasting (MUACZ <−3) | 55/153 (35.9) | 14/153 (9.2) | <0.001 | – | – | – | – | – | – |
|  |  |  |  |  |  |  |  |  |  |
| Values are n/N (%), where N is the number of children with a valid measurement for that index within the age band. p-values from Fisher's exact test comparing children with CP and controls within each age band. | | | | | | | | | |
| Indices are reported only over the ages for which they are applicable: weight-for-height and MUAC-for-age below 60 months, weight-for-age below 120 months, and height-for-age and BMI-for-age across the full age range. Cells outside these ranges are shown as –. | | | | | | | | | |
| Age band sizes: 24–59 months, 155 CP and 155 controls; 60–119 months, 67 CP and 67 controls; ≥120 months, 38 CP and 36 controls. | | | | | | | | | |

**Table S5.** Food groups consumed by children with cerebral palsy and matched controls, by caregiver report

| **Food group** | **CP (n=251)** | **Controls (n=246)** |
| --- | --- | --- |
| Cereals and cereal products | 247 (98.4) | 234 (95.1) |
| Vegetables | 187 (74.5) | 194 (78.9) |
| Fats and oils | 134 (53.4) | 174 (70.7) |
| Pulses / legumes / nuts and seeds | 122 (48.6) | 85 (34.6) |
| Sugars / honey and commercial juices | 109 (43.4) | 88 (35.8) |
| Fish and sea foods | 98 (39.0) | 131 (53.3) |
| Milk and milk products | 51 (20.3) | 12 (4.9) |
| Fruits | 43 (17.1) | 55 (22.4) |
| Roots and tubers | 29 (11.6) | 52 (21.1) |
| Eggs | 16 (6.4) | 30 (12.2) |
| Meats and poultry | 12 (4.8) | 13 (5.3) |
| Breast milk | 2 (0.8) | 2 (0.8) |
| Miscellaneous | 2 (0.8) | 1 (0.4) |

Assessed using an open question on what the child eats on a typical day, with responses coded against the listed food groups. Percentages calculated on children with complete responses (251 children with CP, 246 controls); data were missing for 9 children with CP (3.5%) and 13 controls (5.0%).
